# Knowledge and Willingness to Utilize Hepatitis B Preventive Measures among Pregnant Women in Ado-Ekiti, Southwest, Nigeria

**DOI:** 10.1155/2023/9168038

**Published:** 2023-11-16

**Authors:** Idowu Pius Ade-Ojo, Adefunke Olarinre Babatola, Temitope Olumuyiwa Ojo, Ezra Olatunde Ogundare, Tosin Agbesanwa, Adewuyi Temidayo Adeniyi, Omolola Alao, Oladele Simeon Olatunya, Joseph Olusesan Fadare

**Affiliations:** ^1^Department of Obstetrics and Gynaecology, Ekiti State University, Ado-Ekiti, Ekiti State, Nigeria; ^2^Department of Obstetrics and Gynaecology, Ekiti State University Teaching Hospital, Ado-Ekiti, Ekiti State, Nigeria; ^3^Department of Paediatrics, Ekiti State University, Ado-Ekiti, Ekiti State, Nigeria; ^4^Department of Paediatrics, Ekiti State University Teaching Hospital, Ado-Ekiti, Ekiti State, Nigeria; ^5^Department of Community Health, Obafemi Awolowo University, Ile-Ife, Osun State, Nigeria; ^6^Department of Family Medicine, Ekiti State University, Ado-Ekiti, Ekiti State, Nigeria; ^7^Department of Pharmacology and Therapeutics, Ekiti State University, Ado-Ekiti, Ekiti State, Nigeria

## Abstract

**Introduction:**

Mother-to-child transmission remains an important mode of transmission of hepatitis B infection particularly in endemic areas. The knowledge and practices of pregnant women about mother-to-child transmission (MTCT) of hepatitis B virus (HBV) may influence the uptake of strategies to reduce mother-to-child transmission of infection.

**Objectives:**

This study assessed the knowledge and willingness to uptake hepatitis B virus infection preventive services among pregnant women in Ado-Ekiti, Nigeria.

**Methods:**

This was a cross-sectional study that involved 373 pregnant women at the Ekiti State University Teaching Hospital (EKSUTH) and Maternal Child Specialist Clinics, Ado-Ekiti, Nigeria. A structured questionnaire was used to assess their knowledge, practices, and perceptions about MTCT of hepatitis B infection.

**Results:**

Only 52.5% (196) of the respondents had good knowledge, although the majority 290 (77.7%) had heard of hepatitis B infection prior to the survey. Only 147 (39.4%) of the respondents had ever had hepatitis B screening. More persons with professional jobs had good knowledge about hepatitis B infection compared with other occupations (*p* < 0.001). However, more respondents aged 30–34 years had poor knowledge about hepatitis B infection compared with other age groups (*p* = 0.045). Respondents with good knowledge about hepatitis B infection were willing to uptake hepatitis B infection prevention services (*p* < 0.001)

**Conclusion:**

This study showed that respondents with professional jobs had good knowledge about hepatitis B infection and those who had good knowledge about the infection were willing to utilize hepatitis B preventive measures. Awareness of MTCT of HBV did not translate into good practice as only few respondents had screened for hepatitis B. There is a need to intensify education about modes of transmission of hepatitis B infection with an emphasis on promoting good preventive practices.

## 1. Introduction

Chronic hepatitis B virus (HBV) infection is a major public health problem. As at 2019, about 296 million persons worldwide were infected and about 1.5 million people acquire the infection annually. These persons mostly reside in low and middle-income countries [1, 2]. The prevalence of HBV infection varies throughout the world, and it is highly endemic in developing countries: South East Asia, China, sub-Saharan Africa, and the Amazon Basin where at least 8% of the population are chronic carriers of hepatitis B virus [3]. Nigeria also has a significant burden of HBV infection; for instance, a systematic analysis done by Ajuwon et al. showed the pooled prevalence of HBV infection in Nigeria to be 9.5% [4].

In Nigeria, the prevalence of HBV infection varies from region to region with the North western region having the highest burden [4]. In Ekiti state, prevalence rates of 3.9% and 6.2% were reported amongst pregnant women and adults, respectively [5, 6]. Also, amongst children and adolescents, varying seroprevalences have been reported from different parts of Nigeria [7–13].

The impact of HBV infection is devastating for individuals, families, communities, and the world at large. HBV infection was responsible for 820,000 deaths worldwide in 2019 [1]. Chronic HBV infection is also an important and major risk factor for liver cirrhosis and hepatocellular carcinoma [1, 14].

Hepatitis B virus (HBV) is mostly transmitted through contact with infected body fluids, and the predominant mode of transmission is believed to be related to the endemicity of HBV infection in the particular area [3]. Nigeria, being an area with high hepatitis B virus endemicity, a major mode of transmission, is mother to child transmission of the virus [1]. Other modes of transmission are sexual and parenteral/percutaneous transmission [3]. Therefore, reducing mother to child transmission of hepatitis B virus may reduce the global and local prevalence of chronic hepatitis B infections. It is also well known that mother to child transmission causes the highest chronic carrier rate of greater than 85% [1, 15], and this risk is reduced by 90% with HBV vaccine and hepatitis B immunoglobulin administration within 12–24 hours of birth [15].

Hence, to achieve the World Health Organization's target of reducing HBV infection to 0.1% by 2030 [16], one of the main areas of focus should be prevention of mother to child transmission of hepatitis B virus. Available strategies to prevent or reduce mother to child transmission of HBV include hepatitis B vaccination, administration of hepatitis B immunoglobulin (HBIG) to newborn at birth, and use of antiviral prophylaxis in pregnancy. Hepatitis B vaccination is freely available for newborn and infants as it is incorporated into the routine immunization schedule of the National Programme on Immunization. HBIG and antivirals are readily available in the country for a fee. Currently, in Nigeria, the coverage for many of the HBV preventive strategies is suboptimal; for example, hepatitis B vaccination coverage among children is low; according to the 2018 National Demographic Health Survey, only 52% of children aged 12–23 months received hepatitis B vaccine at birth [17].

Having information on the knowledge and practices of pregnant women about HBV infection particularly mother-to-child transmission will identify gaps which stakeholders and policy makers could address towards achieving the desired goal of reducing/eliminating HBV infection [18]. Reports from previous studies conducted in Nigeria and outside on evaluation of the level of knowledge of and practices of pregnant women about HBV infection have been inconsistent [19–23]. The authors are not aware of any study that assessed knowledge particularly of prevention of mother to child transmission of hepatitis B virus among pregnant women in Ekiti State, Nigeria. Therefore, this study aimed to assess knowledge and willingness to uptake HBV infection preventive services among pregnant women in Ado-Ekiti, Nigeria.

## 2. Methods

### 2.1. Study Design and Setting

A cross-sectional study conducted at the Antenatal Care Clinics of the Ekiti State University Teaching Hospital (EKSUTH) and Maternal Child Specialist Clinics. Both health facilities are located in Ado-Ekiti, the capital city of Ekiti State, Nigeria. The Ekiti State University Teaching Hospital was established in 2008 and serves as a referral centre for 3 specialist hospitals, 18 secondary health facilities, and over 150 primary health care facilities owned by the Ekiti State Government. The Obstetrics and Gynaecology Department operates the Antenatal Clinic four days a week (Mondays, Wednesdays, Thursdays, and Fridays) with an average of 400 pregnant women attending monthly. The Maternal Child Specialist Clinics (MCSC) is a private-owned health facility. It is one of the high-volume hospitals in Ado-Ekiti and conducts antenatal clinic once weekly (Tuesdays) with an average of 100 pregnant women attending the clinic monthly. The two health facilities have separate booking clinic days that are not part of the antenatal clinic days. The study was conducted from August to October 2019.

### 2.2. Study Population

Pregnant women who have booked their pregnancies and receiving antenatal care at the Ekiti State University Teaching Hospital and Maternal Child Specialist Clinics.

### 2.3. Sample Size Determination

The sample size was calculated using online software Raosoft sample size calculator (https://www.raosoft.com/samplesize.html) using the following assumptions; 5% margin of error, confidence level of 95%, response distribution of 50%, and estimated total population of pregnant women in Ado-Ekiti of 20,000. The sample size obtained was 377, and a 5% (19) attrition rate was added to the sample size giving a sum of 396. A total of 400 questionnaires were administered.

### 2.4. Sampling Method

A convenience sampling was used: all consecutive attendees of the antenatal clinics of the two health facilities who gave consent to participate in the study were recruited.

#### 2.4.1. Inclusion Criterion

The inclusion criterion was every pregnant woman attending the ante-natal clinics who gives consent to be a part of the study.

#### 2.4.2. Exclusion Criteria

Exclusion criteria were pregnant women who have not registered or undergone booking clinic experience and women who were acutely ill and unable to respond to the interview.

### 2.5. Study Instrument

The questionnaire was adapted from similar studies [19–21, 23], self- and interviewer-administered for the uneducated. It had three sections. The first section (Section A) had questions on socio-demographics which include age, highest education attained, occupation, marital status, and tribe. The second section (Section B) had questions on awareness about hepatitis B infection, hepatitis B vaccine, hepatitis B immunoglobulin, history of hepatitis B screening, willingness to be screened, and willingness to allow child to receive hepatitis B vaccine and immunoglobulin if required. The third section (Section C) had eleven statements on knowledge of transmission of HBV and ways of preventing its transmission using a 5-point Likert scale responses from strongly agree to strongly disagree. It comprised 9 positive statements and 2 negative statements. A score was assigned to each of the responses; strongly agree (5 points), agree (4 points), neutral (3 points), disagree (2 points), and strongly disagree (1 point) for positive statements, and the scores were reversed for negative statements. The content validity of the questionnaire was conducted by experts in the field of paediatric gastroenterology, neonatology, infectious diseases, and obstetrics. The questionnaire was pretested among forty pregnant women in another hospital that was not part of the study, and ambiguous/unclear questions were removed. The questionnaire has a high reliability with the Cronbach alpha coefficient of 0.85.

### 2.6. Data Analysis

Data were analyzed using IBM SPSS version 25 (Statistical Package for Social Sciences). Categorical variables such as marital status of pregnant women were summarized using frequencies and percentages. The mean and standard deviation were used to summarize continuous variables, e.g., age of respondents. Overall knowledge about hepatitis B virus transmission and prevention was assessed using 11 questions. The median score was used to categorize knowledge into poor or good. The median score was 40; hence, scores of 40 and above were classified as good knowledge. The chi square test was used to assess the association between two categorical variables such as age groups and respondents' knowledge about hepatitis B infection. Statistical significance was set at *p* < 0.05. Results are presented in tables and chart.

## 3. Results

Out of 400 questionnaires that were distributed, 384 questionnaires were returned giving a response rate of 96.0%. Eleven questionnaires had incomplete information, hence removed from the analysis. Therefore, a total of 373 questionnaires were analyzed.

### 3.1. Socio-Demographic Characteristics of the Respondents

The age group with the highest frequency was 30–34 years (Mean ± SD: 31.0 ± 5.3 years), 113 (30.3%) were 25–29 years, and 27 (7.2%) were 40 years or more. Three hundred and thirty-nine (90.9%) of them had tertiary education, 368 (98.7%) were married, and 150 (40.2%) were traders. The respondents were predominantly Yoruba (289; 77.5%) (Table 1).

### 3.2. Awareness/Knowledge of Hepatitis B Virus and Willingness to Uptake Hepatitis B Infection Preventive Services

**Figure 1:**
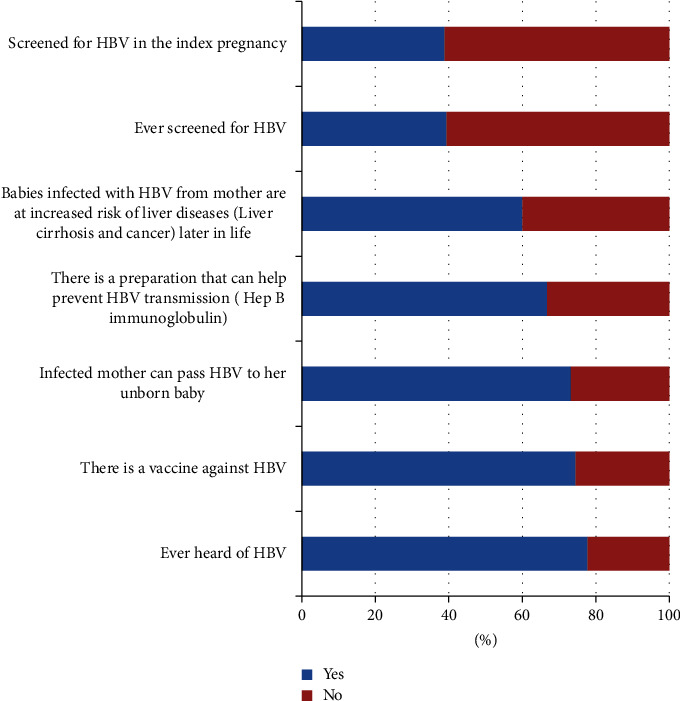
Awareness of hepatitis B and practice of HBV screening among respondents.

Overall, 196 (52.5%) respondents had good knowledge. Majority of the respondents had heard of hepatitis B infection, 290 (77.7%), while 278 (74.5%) knew there is a vaccine against hepatitis B infection, and 273 (73.2%) respondents indicated that an infected mother could infect her newborn with the virus. About two-third, 249 (66.8%) of the respondents knew that there is a preparation (hepatitis B immunoglobulin) that can help prevent transmission of hepatitis B infection from mother to baby. Sixty percent of the respondents knew that babies infected with hepatitis B virus from mothers have an increased risk of developing liver diseases (liver cirrhosis and cancer) later in life. However, only 147 (39.4%) had ever had hepatitis B screening and most (145/147) of those that had done hepatitis B screening had the screening during the index pregnancy (Figure 1).

**Figure 2:**
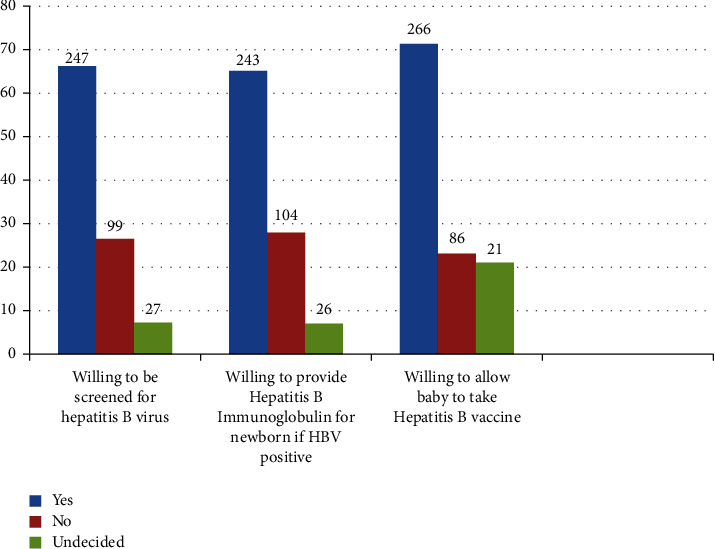
Respondents' willingness to uptake hepatitis B preventive services.

About two-third (66.2%) of the respondents were willing to have the hepatitis B screening test. Majority 243 (65.1%) of the respondents were willing to provide hepatitis B immunoglobulin for their newborn if there is a need. Two hundred and sixty-six (71.3%) respondents were willing to allow their babies to take hepatitis B vaccine (Figure 2).

### 3.3. Respondents' Perceptions about Modes of Transmission and Prevention of Mother to Child Transmission of Hepatitis B Virus

Slightly more than one-half (59.8% and 56.6%) of the respondents agreed that hepatitis B virus can be transmitted through unprotected sexual intercourse, and there is an increased risk of contracting hepatitis B infection from unsafe injections, respectively. Majority 270 (72.4%) of the respondents agreed that hepatitis B virus can be transmitted through transfusion of infected blood. Nearly one-half (49.3%) of the respondents neither agreed nor disagreed to the statement that hepatitis B virus can be transmitted from animals to humans. Also, majority 249 (66.7%) of the respondents agreed that hepatitis B exposed babies require hepatitis B vaccine to prevent them from being infected. One hundred and forty-five (38.9%) of the respondents neither agreed nor disagreed to whether hepatitis B vaccine was free for all babies and 204 (54.7%) did not know whether hepatitis B immunoglobulin is free or not (Table 2).

### 3.4. Association between Socio-Demographic Characteristics of Respondents and Level of Knowledge about Hepatitis B Infection

There was statistically significant association between the level of knowledge about hepatitis B infection and age of respondents; more respondents aged 30–34 years had poor knowledge when compared with the other age brackets (*p*=0.045). However, more respondents who were professionals had good knowledge about hepatitis B infection compared with other occupations (*p* < 0.001). There was no significant association between the level of knowledge about hepatitis B infection and other socio-demographic characteristics (tribe, religion, marital status, and the highest level of education) (Table 3)

### 3.5. Association between Respondents' Level of Knowledge and Willingness to Uptake Hepatitis B Infection Preventive Services

There was significant association between the level of knowledge about hepatitis B infection and willingness to uptake preventive services. More respondents who had good knowledge were willing to have HBV screening, allow their babies to have HBV vaccine, and provide hepatitis B immunoglobulin for their child (*p* < 0.001) (Table 4).

## 4. Discussion

Pregnant women remain major stakeholders in achieving the WHO goal of eliminating hepatitis by year 2030 as mother to child transmission of HBV is a major route of transmission particularly in hepatitis B infection endemic countries like Nigeria. This study assessed the knowledge of practices and perceptions about hepatitis B among pregnant women in Ado-Ekiti, Nigeria. It also assessed the willingness of pregnant women to utilize hepatitis B infection prevention services. In all, barely one-half of the respondents had overall good knowledge about hepatitis B infection although majority of them were aware of hepatitis B virus and modes of preventing transmission from mother to child and were willing to uptake prevention services. However, as high as 40% of respondents did not know some important ways by which hepatitis B infection could be contracted and only few of them had ever screened for hepatitis B.

That barely one-half (52.5%) of our respondents had overall good knowledge about mother to child transmission (vertical transmission) of hepatitis B infection, and its modes of prevention are disturbing because pregnant women are major stakeholders in combating the burden of this infection considering the fact that vertical transmission is an important route of transmission of this infection which also causes the most chronic carrier rate [1]. Chronic carriage of hepatitis B virus (chronic hepatitis) is associated with an increased risk of developing liver cirrhosis and liver carcinoma [24, 25].

In this study, although there was a high level of awareness about hepatitis B infection amongst the respondents as majority (78%) of them were aware of the infection, only about one-half of them had overall good knowledge, and this may suggest a need to intensify education about hepatitis B infection and put structures in place that will encourage routine screening of pregnant women and vaccination as and when due. Having majority of our respondents aware of hepatitis B infection is comparable to 64.4% reported by Odelola et al. from a similar study conducted in Sagamu, Ogun State Nigeria [21]. However, a similar study by Idowu et al. in Oyo State, Nigeria, observed that a lower proportion (51.8%) of their respondents were aware of hepatitis B virus infection [22]. Idowu et al. conducted their study among pregnant women at a primary health care centre and ours was at a tertiary hospital and a private facility where more of our respondents had tertiary education (90.9% versus 54.8%).

In this study, about 7 out of 10 respondents knew that an infected mother could infect her new-born with the virus. This finding is in consonance with the report of a study conducted in Ghana [23]. Our finding that about 70% of our respondents were aware of mother-to-child transmission of the virus may explain why majority (>60%) of them were willing to uptake hepatitis B infection prevention services (hepatitis B screening, hepatitis B immunoglobulin, hepatitis B vaccine). This emboldens the need to make hepatitis B prevention services readily accessible and affordable to pregnant women by policy makers and health care providers. However, a similar study from Ogun State, Nigeria, found that only 47.4% of their respondents knew about mother-to-child transmission of hepatitis B infection and only about one-third of their respondents were willing to uptake hepatitis B prevention services [21]. This difference between our study and the study from Ogun State, Nigeria, may be due to the educational status of our study participants. Majority (90.9%) of our respondents had tertiary education, while only 30% of theirs had tertiary education. It is well known that the level of formal education may have influence on health seeking behaviour.

In this study, we observed that more than 40% of our respondents did not know that hepatitis B virus could be transmitted through sexual intercourse and unsafe injections. This is a cause for concern as engagements in these risky behaviours due to ignorance may continue to fuel transmission of the infection and stall/impede efforts towards its elimination. Previous similar studies have also reported having many of their respondents being unaware of sexual intercourse and unsafe injections as modes of transmission of hepatitis B infection [21, 26, 27].

Only 39.4% (147) of our respondents had ever had hepatitis B screening. Many previous studies have reported a similar observation. These include 14.1% by Gebrecherkos et al. in northwest Ethiopia [20], 41.7% by Nsiah et al. in Kumasi, Ghana [19], and 8.3% by Katamba et al. in northern Uganda [28]. At variance with our finding is a report from Vietnam which stated that 62.4% of the pregnant women in their study had hepatitis B screening during their recent pregnancy [29]. Our study finding that only about two-fifth of the respondents ever had hepatitis B screening is worrisome because the high level of awareness (78%) among them does not seem to translate to practice though the suboptimal level of knowledge about hepatitis B infection reported in this study may contribute to this poor level of practice. It is quite concerning considering the fact that routine hepatitis B screening during antenatal period has been adopted by Nigeria as part of interventions to eliminate mother-to-child transmission of hepatitis B virus [30]. This observation raises the need for studies focused on reasons or barriers to uptake of hepatitis B screening services during the ante-natal period among women in Nigeria. The difference in the hepatitis B screening rate reported in our study and the Vietnamese study is probably due to high attention on hepatitis B infection in Vietnam occasioned by its high prevalence and burden in the country [31].

It is worrisome that only 2 (0.54%) of out of all our respondents had hepatitis B screening outside the current pregnancy period. This could reflect accessibility and availability of hepatitis B screening services outside the antenatal care services. It could also be due to suboptimal knowledge of hepatitis B infection and its complications [32, 33].

In this study, there was significant association between the level of knowledge about hepatitis B infection and age of respondents; more of those aged 30–34 years had poor knowledge compared to other age groups. This finding is a cause for worry because majority of our study participants were in this age group, and it is expected that most of these pregnant women at this age would have had previous pregnancy experience and thus should have had access to this information from ante-natal care services. Also, we observed significant association between the level of knowledge and occupation. More professionals had good knowledge about hepatitis B infection. It may imply that they are more exposed to sources of information about hepatitis B infection. A similar study conducted in Ghana reported a similar observation that majority of pregnant women who had excellent knowledge about hepatitis B infection worked in the formal sector [34].

This study showed no association between the level of knowledge about hepatitis B infection and respondents' highest level of education, and this contradicts previous reports among similar population [19, 21, 34]. The reason for this is not clear. This study observed the suboptimal level of knowledge about hepatitis B infection in spite of the high level of awareness of the infection among pregnant women. Although majority of the pregnant women were aware of the infection, it did not translate into good practice. However, most of them were willing to uptake hepatitis B infection preventive services, and this is commendable.

### 4.1. Study Limitations

Being a questionnaire-based study, there is possibility of some bias associated with self-reporting. However, to minimize this, the participant's information section stated the purpose of the study (academic) and requested for honest answers. Also, the questionnaire had no name or any means of identification. We acknowledge that some important questions were omitted; we did not ask questions on the respondents' source of information about hepatitis B infection, we did not ask for parity, and we did not ask for reasons why those that have not had hepatitis B screening have not had it. Also, the convenience sampling method used may also have excluded some participants who presented when the researchers were not available at the clinics. Despite these limitations, this study provided information on the level of awareness/knowledge of hepatitis B infection, practice of hepatitis B screening, and willingness to uptake hepatitis B preventive services among pregnant women in the study locality.

## 5. Conclusion

This study showed that respondents who were professionals had good knowledge about hepatitis B infection and those who had good knowledge about the infection were willing to utilize hepatitis B preventive measures. Ensuring that women attain high socioeconomic status may be a measure in addressing the burden of hepatitis B transmission. The study also observed that despite the high level of awareness about hepatitis B infection and the fact that majority (73%) were aware of mother to child transmission of HBV, it did not translate into good practice as only a few (39.4%) of the respondents had hepatitis B screening. Hence, there is a need to intensify education about hepatitis B infection with an emphasis on the mode of transmission at various fora and media of communication. Furthermore, measures should be instituted to encourage good practices related to prevention of mother to child transmission of hepatitis B infection which include ensuring national policy on hepatitis B screening for all pregnant women as part of ante-natal care and subsidizing or providing ante-natal care services free of cost.

## Figures and Tables

**Table 1:** Sociodemographic characteristics of respondents.

Characteristics	Frequency	Percentage (%)
*Age in years*		
18–24	31	8.3
25–29	113	30.3
30–34	149	39.9
35–39	53	14.2
40 yrs or more	27	7.2
Mean, SD: 31.0 ± 5.3 yrs		

*Highest education attained*		
No formal education	2	0.5
Primary	3	0.8
Secondary	29	7.8
Tertiary	339	90.9

*Marital status*		
Married	368	98.7
Single	4	1.1
Divorced	1	0.3

*Occupation*		
Professional	36	9.7
Trader/business	150	40.2
Artisan	23	6.2
Unemployed	40	10.7
Farmer	3	0.8
Others	4	1.1

*Religion*		
Christianity	340	91.2
Islam	33	8.8

*Tribe*		
Igbo	39	10.5
Hausa	33	8.8
Yoruba	289	77.5
Others	12	3.2

**Table 2:** Respondents' perceptions about modes of transmission and prevention of mother to child transmission of hepatitis B virus.

Variables	Strongly agree *n* (%)	Agree *n*(%)	Neutral *n*(%)	Disagree *n*(%)	Strongly disagree *n* (%)
Hepatitis B virus can be transmitted through unprotected sexual intercourse	111 (29.8)	112 (30.0)	101 (27.1)	41 (11.0)	8 (2.1)
Hepatitis B virus can be transmitted through transfusion of infected blood	132 (35.4)	138 (37.0)	83 (22.3)	17 (4.6)	3 (0.8)
Hepatitis B virus is transmitted from animals to human beings	33 (8.8)	68 (18.2)	184 (49.3)	77 (20.6)	11 (2.9)
Hepatitis B virus is present in significant amount in breastmilk	76 (20.4)	89 (23.9)	169 (45.3)	33 (8.8)	6 (1.6)
There is an increased risk of contracting hepatitis B infection from unsafe injections	94 (25.2)	117 (31.4)	131 (35.1)	27 (7.2)	4 (1.1)
Babies delivered to mothers with hepatitis B virus infection are termed hepatitis B exposed	85 (22.8)	142 (38.1)	122 (32.7)	22 (5.9)	2 (0.5)
Hepatitis B exposed babies require hepatitis b vaccine to prevent them from being infected	128 (34.3)	121 (32.4)	111 (29.8)	12 (3.2)	1 (0.30)
Hepatitis B exposed babies require hepatitis B immunoglobulin to prevent them from being infected	128 (34.3)	112 (30.0)	117 (31.4)	13 (3.5)	3 (0.8)
Hepatitis B vaccine should be given to babies at birth	146 (39.1)	106 (28.4)	105 (28.2)	10 (2.7)	6 (1.6)
Hepatitis B vaccine is free for all babies	115 (30.8)	89 (23.9)	145 (38.9)	18 (4.8)	6 (1.6)
Hepatitis B immunoglobulin is not free	60 (16.1)	57 (15.3)	204 (54.7)	40 (10.7)	12 (3.2)

**Table 3:** Association between socio-demographic characteristics of respondents and knowledge about hepatitis B infection.

Variable	Good knowledge (*N* = 196) *n* (%)	Poor knowledge (*N* = 177) *n* (%)	Total (*N* = 373) *n* (%)	Chi-square, df
*Age groups (years)*				
18–24	17 (54.8)	14 (45.2)	31 (100.0)	
25–29	68 (60.2)	45 (39.8)	113 (100.0)	9.728, 4
30–34	64 (43.0)	85 (57.0)	149 (100.0)	**P = 0.045**
35–39	30 (56.6)	23 (43.4)	53 (100.0)	
>40	17 (63.0)	10 (37.0)	27 (100.0)	

*Tribe*				
Hausa	12 (36.4)	21 (63.6)	33 (100.0)	5.090, 3
Igbo	23 (59.0)	16 (41.0)	39 (100.0)	**P = 0.165**
Yoruba	153 (52.9)	136 (47.1)	289 (100.0)	
Others	8 (33.7)	4 (66.7)	12 (100.0)	

*Religion*				
Islam	16 (48.5)	17 (51.5)	33 (100.0)	0.240, 1
Christianity	180 (52.9)	160 (47.1)	340 (100.0)	**P = 0.625**

*Marital status*				
Single/divorced	3 (60.0)	2 (40.0)	5 (100.0)	0.113, 1
Married	193 (52.4)	175 (47.6)	368 (100.0)	**P = 0.737**

*Highest level of education*				
None	1 (50.0)	1 (50.0)	2 (100.0)	
Primary	1 (33.3)	2 (66.7)	3 (100.0)	4.652, 3
Secondary	10 (34.5)	19 (65.5)	29 (100.0)	**P = 0.199**
Tertiary	184 (54.3)	155 (45.7)	339 (100.0)	

*Occupation*				
Civil servant	67 (57.3)	50 (42.7)	117 (100.0)	24.537, 6
Professional	29 (80.6)	7 (19.4)	36 (100.0)	**P < 0.001**
Trader/business	62 (41.3)	88 (58.7)	150 (100.0)	
Artisan	9 (39.1)	14 (60.9)	23 (100.0)	
Unemployed	26 (65.0)	14 (35.0)	40 (100.0)	
Farmer	1 (33.3)	2 (66.7)	3 (100.0)	
Others	2 (50.0)	2 (50.0)	4 (100.0)	

*P* < 0.001 should be in bold font. df, degree of freedom.

**Table 4:** Association between respondents' level of knowledge and willingness to uptake hepatitis B prevention services.

Variable	Good knowledge (*N* = 196) *n* (%)	Poor knowledge (*N* = 177) *n* (%)	Total (*N* = 373) *n* (%)	Chi-square, df
*Are you willing to be screened for HBV*				
Yes	163 (66.0)	84 (34.0)	247 (100.0)	53.711, 2
No	24 (24.2)	75 (75.8)	99 (100.0)	**P < 0.001**
Undecided	9 (33.3)	18 (66.7)	27 (100.0)	

*Willing you be willing to allow your baby take HBV vaccine*				
Yes	167 (62.8)	99 (37.2)	266 (100.0)	39.363, 2
No	22 (25.6)	64 (74.4)	86 (100.0)	**P < 0.001**
Undecided	7 (33.3)	14 (66.7)	21 (100.0)	

*Willing you be willing to provide HBIG for your baby if there is a need*				
Yes	159 (65.4)	84 (34.6)	243 (100.0)	46.917, 2
No	28 (26.9)	76 (73.1)	104 (100.0)	**P < 0.001**
Undecided	9 (34.6)	17 (65.4)	26 (100.0)	

*P* values in bold fonts indicate statistical significance. df, degree of freedom.

## Data Availability

The data used for this study are in the results section of this article.

## Abbreviations

EKSUTH: Ekiti State University Teaching Hospital
HBIG: Hepatitis B immunoglobulin
HBV: Hepatitis B virus
MTCT: Mother to child transmission
SD: Standard deviation
WHO: World Health Organization

## Supplementary Materials

Supplementary Materials Data collection instrument: a questionnaire was used for data collection. Supplementary Table 1: association between socio-demographic characteristics of respondents and the level of knowledge about hepatitis B infection disaggregated by place of receiving ante-natal care. Supplementary Table 2: association between the respondents' level of knowledge and willingness to uptake hepatitis B prevention services by place of receiving ante-natal care.

## References

[1] World Health Organization (2022). Hepatitis B. https://www.who.int/news-room/fact-sheets/detail/hepatitis-b.

[2] World Health Organization (2017). Guidelines on hepatitis B and C testing. https://www.who.int/publications-detail-redirect/9789241549981.

[3] Hou J., Liu Z., Gu F. (2005). Epidemiology and prevention of hepatitis B virus infection. *International Journal of Medical Sciences*.

[4] Ajuwon B. I., Yujuico I., Roper K., Richardson A., Sheel M., Lidbury B. A. (2021). Hepatitis B virus infection in Nigeria: a systematic review and meta-analysis of data published between 2010 and 2019. *BMC Infectious Diseases*.

[5] Ajayi A. O., Ade-ojo I. P., Ajayi E. A., Adegun P. T., Ojo Aduloju O. P. (2013). Seroprevalence of hepatitis B infection in pregnant women at the Ekiti state university teaching hospital, ado-ekiti, southwest Nigeria. *African Journal of Internal Medicine*.

[6] Thompson J. A., Festus A. O., Festus O. A., Olalekan I. O., Funmilayo J. A. (2015). Hepatitis B virus (HbV) and syphilis co-infections among the people of Ekiti, south-west, Nigeria. *International Journal of Medical Research and Health Sciences*.

[7] David O. M., Oluduro A. O., Ariyo A. B., Ayeni D., Famurewa O. (2013). Sero-epidemiological survey of hepatitis B surface antigenaemia in children and adolescents in Ekiti State. *Journal of Public Health and Epidemiology*.

[8] Babatola A. O., Olatunya O. S., Faboya A. O. (2020). Hepatitis B and C Infections among pediatric patients with sickle cell disease at a tertiary hospital in Nigeria. *Archives of Pediatric Infectious Diseases*.

[9] Sadoh A. E., Ofili A. (2014). Hepatitis B infection among Nigerian children admitted to a children’s emergency room. *African Health Sciences*.

[10] Uleanya N. D., Obidike E. O. (1970). Prevalence and risk factors of hepatitis B virus transmission among children in Enugu, Nigeria. *Nigerian Journal of Paediatrics*.

[11] Lawal M. A., Adeniyi O. F., Akintan P. E., Salako A. O., Omotosho O. S., Temiye E. O. (2020). Prevalence of and risk factors for hepatitis B and C viral co-infections in HIV infected children in Lagos, Nigeria. *PLoS One*.

[12] Ikobah J., Okpara H., Elemi I., Ogarepe Y., Udoh E., Ekanem E. (2016). The prevalence of hepatitis B virus infection in Nigerian children prior to vaccine introduction into the National Programme on Immunization schedule. *The Pan African Medical Journal*.

[13] Jibrin B., Jiya N. M., Ahmed H. (2014). Prevalence of Hepatitis B surface antigen in children with sickle cell anemia. *Sahel Medical Journal*.

[14] Tong M. J., Blatt L. M., Tyson K. B., Kao V. W. C. (2006). Death from liver disease and development of hepatocellular carcinoma in patients with chronic Hepatitis B Virus Infection: a prospective study. *Gastroenterology and Hepatology*.

[15] Stevens C. E., Toy P., Kamili S. (2017). Eradicating hepatitis B virus: the critical role of preventing perinatal transmission. *Biologicals*.

[16] World Health Organization WHO releases first-ever global guidance for country validation of viral hepatitis B and C elimination. https://www.who.int/news/item/25-06-2021-who-releases-first-ever-global-guidance-for-country-validation-of-viral-hepatitis-b-and-c-elimination.

[17] NPC Engineering Industries (2019). Nigeria demographic and health survey 2018-final report. https://dhsprogram.com/publications/publication-fr359-dhs-final-reports.cfm.

[18] Eleje G. U., Akaba G. O., Mbachu I. I. (2021). Pregnant women’s hepatitis B vaccination coverage in Nigeria: a national pilot cross-sectional study. *Therapeutic Advances in Vaccines and Immunotherapy*.

[19] Nsiah I., Danquah C. B., Anto E. O. (2020). Factors associated with knowledge, attitude and practice towards hepatitis B infection among pregnant women attending antenatal clinic in the Kumasi Metropolis, Ghana: a multi-centre hospital-based cross-sectional study. *PAMJ-One Health*.

[20] Gebrecherkos T., Girmay G., Lemma M., Negash M. (2020). Knowledge, attitude, and practice towards hepatitis B virus among pregnant women attending antenatal care at the university of gondar comprehensive specialized hospital, northwest Ethiopia. *International Journal of Hepatology*.

[21] Odelola O. I., Akadri A. A., Shorunmu T. O. (2020). Hepatitis B virus infection: knowledge of antenatal attendees in a tertiary hospital. *Nigerian Journal of Medicine*.

[22] Idowu A., Israel O. K., Aremu O. A., Akinwumi A. F. (2019). Seroprevalence and determinants of hepatitis B viral status in pregnant women attending antenatal clinics in an urban community of Oyo state, South-West Nigeria. *International Journal Of Community Medicine And Public Health*.

[23] Dun-Dery F., Adokiya M. N., Walana W., Yirkyio E., Ziem J. B. (2017). Assessing the knowledge of expectant mothers on mother-to-child transmission of viral hepatitis B in Upper West region of Ghana. *BMC Infectious Diseases*.

[24] Pan C. Q., Zhang J. X. (2005). Natural history and clinical consequences of Hepatitis B Virus infection. *International Journal of Medical Sciences*.

[25] Shimakawa Y., Yan H. J., Tsuchiya N., Bottomley C., Hall A. J. (2013). Association of early age at establishment of chronic hepatitis B infection with persistent viral replication, liver cirrhosis and hepatocellular carcinoma: a systematic review. *PLoS One*.

[26] Dagnew M., Million Y., Destaw B., Adefris M., Moges F., Tiruneh M. (2020). Knowledge, attitude, and associated factors towards vertical transmission of Hepatitis B Virus among pregnant women attending antenatal care in tertiary hospitals in Amhara Region, Northwest Ethiopia: a Cross-Sectional Study. *International Journal of Women’s Health*.

[27] Han Z., Yin Y., Zhang Y. (2017). Knowledge of and attitudes towards hepatitis B and its transmission from mother to child among pregnant women in Guangdong Province, China. *PLoS One*.

[28] Katamba P. S., Mukunya D., Kwesiga D., Nankabirwa V. (2019). Prenatal hepatitis B screening and associated factors in a high prevalence district of Lira, northern Uganda: a community based cross sectional study. *BMC Public Health*.

[29] Hang Pham T. T., Le T. X., Nguyen D. T. (2019). Knowledge, attitudes and practices of hepatitis B prevention and immunization of pregnant women and mothers in northern Vietnam. *PLoS One*.

[30] National Aids (2016). National guidelines for the prevention, care and treatment of viral hepatitis B and C in Nigeria. https://www.hepb.org/assets/Uploads/Nigeria-Hepatitis-Guidelines-TX-guidelines.pdf.

[31] World Health Organization WHO calls for increased investment in hepatitis elimination. https://www.who.int/vietnam/news/detail/01-08-2019-who-calls-for-increased-investment-in-hepatitis-elimination.

[32] Hepatitis B Foundation (2020). The journey to hepatitis elimination in Nigeria. https://www.hepb.org/blog/journey-hepatitis-elimination-nigeria/.

[33] Eni A. O., Soluade M. G., Oshamika O. O., Efekemo O. P., Igwe T. T., Onile-Ere O. A. (2019). Knowledge and awareness of hepatitis B virus infection in Nigeria. *Annals of Global Health*.

[34] Kwadzokpui P. K., Akorsu E. E., Abaka-Yawson A., Quarshie S. S., Amankwah S. A., Tawiah P. A. (2020). Prevalence and knowledge of hepatitis B virus infection among pregnant women in the Ningo-Prampram District, Ghana. *International Journal of Hepatology*.

