# Supplementary material for: Knowledge and Willingness to Utilize Hepatitis B Preventive Measures among Pregnant Women in Ado-Ekiti, Southwest, Nigeria
Source: Interdiscip Perspect Infect Dis. 2023 Nov 16;2023:9168038. doi: 10.1155/2023/9168038 (PMC10667044; doi:10.1155/2023/9168038)
Supplement: Supplementary Materials — Data collection instrument: a questionnaire was used for data collection. Supplementary Table 1: association between socio-demographic characteristics of respondents and the level of knowledge about hepatitis B infection disaggregated by place of receiving ante-natal care. Supplementary Table 2: association between the respondents' level of knowledge and willingness to uptake hepatitis B prevention services by place of receiving ante-natal care. [file 9168038.f1.zip › PMTCT OF HEPATITIS B INFECTION QUESTIONNAIRE.doc]

PREVENTION OF MOTHER TO CHILD TRANSMISSION OF HEPATITIS B INFECTION: VIEWS AND PRACTICES OF ANTENATAL CLINIC ATTENDEES IN ADO-EKITI

Dear respondent,

Thank you for participating in this survey. This survey is purely for academic purpose and your participation is voluntary. We would be grateful if you could respond to the questions to the best of your ability. You are free to respond to only questions you can answer. No personal details will appear in any reports generated from this study.

SECTION A

1. Age ______________________________________________________________
2. Tribe: Igbo [	] Hausa [	]	Yoruba [	] others_____________________________
3. Religion: Christianity [ ] Islam [	]	Traditional [ ] others(please state)_____________
4. Highest level of formal education_______________________________________
5. Average monthly family income________________________________________
6. Occupation_________________________________________________________
7. Marital status: Married [ ] Single [ ] Divorced [ ] widowed [ ]
8. Number of children__________________________________________________

SECTION B

9. Have you heard about Hepatitis B virus? Yes [ ] No [	]
10. Are you aware of Hepatitis B virus infection? Yes [ ] No [	]
11. Have you been screened for Hepatitis B virus infection in this pregnancy period?Yes [ ] No [	]
12. Have you been screened for Hepatitis B virus infection before now? Yes [ ] No [	]
13. Can an infected mother pass the virus to her newborn baby? Yes [ ] No [	]
14. Is there a vaccine against Hepatitis B virus infection? Yes [ ] No [	]
15. Is there a preparation (Hepatitis B immunoglobulin) that can help prevent the transmission of hepatitis B infection from mother to baby? Yes [ ] No [	]
16. If yes to question 15, how many times will a child visit the hospital to complete the vaccination for hepatitis B vaccine? A) 1 [ ] B)	2 [	] C) 3 [	] D) 4 [	]	E)	5	[	] F)	6 [	]
17. Are babies infected with Hepatitis B virus from mothers at increased risk of developing liver diseases (liver cirrhosis and cancer) later in life? Yes [ ] No [	]
18. Are you willing to be screened for Hepatitis B virus? Yes [ ] No [ ] Undecided [ ]
19. If you were diagnosed of Hepatitis B virus infection, will you be willing to provide Hepatitis B immunoglobulin for your newborn?Yes [ ] No [	] Undecided [	]
20. If you were diagnosed of Hepatitis B virus infection, will you allow your baby to take Hepatitis B vaccine? Yes [ ] No [	] undecided [	]
21. As part of health education in antenatal clinic, have you ever been spoken to on Hepatitis B infection? Yes [ ] No [	]
22. If no to question 19, will you want the topic to be part of what is discussed during antenatal clinic sessions? Yes [ ] No [	] Undecided [	]

SECTION C

| Statement | Strongly Agree | Agree | Neutral | Disagree | Strongly Disagree |
| --- | --- | --- | --- | --- | --- |
| 1. Hepatitis B virus can be transmitted through unprotected sexual intercourse |  |  |  |  |  |
| 2. Hepatitis B virus can be transmitted through transfusion of infected blood |  |  |  |  |  |
| 3. Hepatitis B virus is transmitted from animals to human beings |  |  |  |  |  |
| 4. Hepatitis B virus is present in significant amount in breastmilk |  |  |  |  |  |
| 5. There is an increased risk of contracting Hepatitis B infection from taking injection from quacks |  |  |  |  |  |
| 6. Babies delivered to mothers with Hepatitis B virus infection are termed Hepatitis B exposed |  |  |  |  |  |
| 7. Hepatitis B exposed babies require hepatitis b vaccine to prevent them from being infected |  |  |  |  |  |
| 8. Hepatitis B exposed babies require hepatitis B immunoglobulin to prevent them from being infected |  |  |  |  |  |
| 9. Hepatitis b vaccine should be given to babies at birth |  |  |  |  |  |
| 10. Hepatitis b vaccine is free for all babies |  |  |  |  |  |
| 11. Hepatitis b immunoglobulin is not free |  |  |  |  |  |

Thank you once again
