# Supplementary material for: Knowledge and Willingness to Utilize Hepatitis B Preventive Measures among Pregnant Women in Ado-Ekiti, Southwest, Nigeria
Source: Interdiscip Perspect Infect Dis. 2023 Nov 16;2023:9168038. doi: 10.1155/2023/9168038 (PMC10667044; doi:10.1155/2023/9168038)
Supplement: Supplementary Materials — Data collection instrument: a questionnaire was used for data collection. Supplementary Table 1: association between socio-demographic characteristics of respondents and the level of knowledge about hepatitis B infection disaggregated by place of receiving ante-natal care. Supplementary Table 2: association between the respondents' level of knowledge and willingness to uptake hepatitis B prevention services by place of receiving ante-natal care. [file 9168038.f1.zip › Supplementary Tables IDID.doc]

**Supplementary Table 1: Association between socio-demographic characteristics of respondents and level of knowledge about hepatitis B infection by place of receiving antenatal care**

| Variable | Good knowledge(N=99)  n(%) | Poor knowledge(N=111)  n (%) | Total (N=210) n(%) | Chi- square, df | Good knowledge(N=97)  n(%) | Poor knowledge(N=66)  n (%) | Total (N=163) n(%) | Chi- square, df |
| --- | --- | --- | --- | --- | --- | --- | --- | --- |
| Public facility | | | | | Private facility | | | |
| **Age groups (years)** |  |  |  | 10.805, 4  **P=0.029** |  |  |  | 0.619, 4  P= 0.961 |
| 18-24 | 6(50.0) | 6 (50.0) | 12(100.0) |  | 11(57.9) | 8(42.1) | 19(100.0) |  |
| 25-29 | 37 (59.7) | 25 (40.3) | 62(100.0) |  | 31(60.8) | 20(39.2) | 51(100.0) |  |
| 30-34 | 32(34.8) | 60 (65.2) | 92(100.0) |  | 32(56.1) | 25(43.9) | 57(100.0) |  |
| 35-39 | 19(52.8) | 17(47.2) | 36(100.0) |  | 11(64.7) | 6(35.3) | 17(100.0) |  |
| >40 | 5(62.5) | 3(37.5) | 8(100.0) |  | 12(63.2) | 7(36.8) | 19(100.0) |  |
| **Tribe** |  |  |  | 4.131, 3  P=0.248 |  |  |  | 2.290, 3  P = 0.514 |
| Hausa | 10 (62.5) | 6(37.5) | 16(100.0) |  | 13(56.5) | 10(43.5) | 23(100.0) |  |
| Igbo | 6(31.6) | 13(68.4) | 19(100.0) |  | 6(42.9) | 8(57.1) | 14(100.0) |  |
| Yoruba | 78(46.7) | 89(53.3) | 167(100.0) |  | 75(61.5) | 47(38.5) | 122(100.0) |  |
| Others | 5(62.5) | 3(37.5) | 8(100.0) |  | 3(75.0) | 1(25.0) | 4(100.0) |  |
| **Religion** |  |  |  | 2.718, 1  P =0.099 |  |  |  | 0.430, 1  P = 0.512 |
| Islam | 4(26.7) | 11(73.3) | 15(100.0) |  | 12(66.7) | 6(33.3) | 18((100.0) |  |
| Christianity | 95(48.7) | 100(51.3) | 195(100.0) |  | 85(58.7) | 60(41.4) | 145((100.0) |  |
| **Marital status** |  |  |  |  |  |  |  | 0.076, 1  P= 0.783 |
| Single/Divorced | 2(66.7) | 1(33.3) | 3(100.0) | 3.141, 1, | 1(50.0) | 1(50.0) | 2(100.0) |  |
| Married | 97(46.9) | 110(43.1) | 207(100.0) | P=0.208 | 96(59.6) | 65(40.4) | 161(100.0) |  |
| **Highest level of education** |  |  |  | 2.964, 2  P=0.227 |  |  |  | 2.598, 3  P =0.458 |
| None | 0(0.0) | 0(0.0) | 0(0.0) |  | 1(50.0) | 1(50.0) | 2(100.0) |  |
| Primary | 1(50.0) | 1(50.0) | 2(100.0) |  | 0(0.0) | 1(100.0) | 1(100.0) |  |
| Secondary | 5(27.8) | 13(72.2) | 18(100.0) |  | 5(45.5) | 6(54.5) | 11(100.0) |  |
| Tertiary | 93(48.9) | 97(51.1) | 190(100.0) |  | 91(61.1) | 58(38.9) | 149(100.0) |  |
| **Occupation** |  |  |  |  |  |  |  |  |
| Civil servant | 42(56.0) | 33(44.0) | 75(100.0) | 19.281,6  **P= 0.004** | 25(59.5) | 17(40.7) | 42(100.0) | 11.949, 6  P= 0.063 |
| Professional | 9(75.0) | 3 (25.0) | 12 (100.0) |  | 20(83.3) | 4(16.7) | 24(100.0) |  |
| Trader/Business | 24(30.8) | 54(69.2) | 78(100.0) |  | 38(52.8) | 34(47.2) | 72(100.0) |  |
| Artisan | 7(36.8) | 12(63.2) | 19(100.0) |  | 2(50.0) | 2(50.0) | 4(100.0) |  |
| Unemployed | 14(63.6) | 8(36.4) | 22(100.0) |  | 12(66.7) | 6(33.3) | 18(100.0) |  |
| Farmer | 1(100.0) | 0(0.0) | 1(100.0) |  | 0(0.0) | 2(100.0) | 2(100.0) |  |
| Others | 2(66.7) | 1(33.3) | 3(100.0) |  | 0(0.0) | 1(100.0) | 1(100.0) |  |

**Supplementary Table 2: Association between respondents’ level of knowledge and willingness to uptake hepatitis B prevention services by place of receiving antenatal care**

| Public facility | | | | | Private facility | | | |
| --- | --- | --- | --- | --- | --- | --- | --- | --- |
| Variable | Good knowledge(N=99)  n(%) | Poor knowledge(N=1111)  n (% | Total (N=210) n(%) | Chi- square, df | Good knowledge(N=97)  n(%) | Poor knowledge(N=66)  n (% | Total (N=163) n(%) | Chi- square, df |
| **Are you willing to be screened for HBV** |  |  |  | 32.907, 2  **P<0.001** |  |  |  |  |
| Yes | 83(61.5) | 52(38.5) | 135(100.0) |  | 80(71.4) | 32(28.6) | 112(100.0) | 21.163,2 |
| No | 10 (17.2) | 48 (82.8) | 58(100.0) |  | 14(34.1) | 27(65.9) | 41(100.0) | **P<0.001** |
| Undecided | 6(35.3) | 11 (64.7) | 17(100.0) |  | 3(30.0) | 7(70.0) | 10(10.0) |  |
| **Willing you be willing to allow your baby take HBV vaccine** |  |  |  | 21.112, 2  **P<0.001** |  |  |  |  |
| Yes | 84 (57.5) | 62(42.5) | 146(100.0) |  | 83(69.2) | 37(30.8) | 120(100.0) | 17.707,2 |
| No | 11(21.6) | 64(78.4) | 51(100.0) |  | 11(31.4) | 24(68.6) | 35(100.0) | **P<0.001** |
| Undecided | 4(30.8) | 14(69.2) | 13(100.0) |  | 3(37.5) | 5(62.5) | 8(100.0) |  |
| **Willing you be willing to provide HBIG for your baby if there is a need** |  |  |  | 27.701, 2  **P <0.001** |  |  |  |  |
| Yes | 77(59.7) | 52(40.3) | 129(100.0) |  | 82(71.9) | 32(28.1) | 114(100.0) | 24.314, 2 |
| No | 16(25.0) | 48(75.0) | 64(100.0) |  | 12(30.0) | 28(70.0) | 40(100.0) | **P<0.001** |
| Undecided | 6 (35.3) | 11(64.7) | 17(100.0) |  | 3(33.3) | 6(66.7) | 6(66.7) |  |
